# Extra axial adult cerebellopontine angle medulloblastoma: An extremely rare site of tumor with metastasis

**DOI:** 10.4103/2152-7806.77178

**Published:** 2011-02-26

**Authors:** Manish Singh, Goutham Cugati, Nigel Peter Symss, Anil Pande, Madabushi Chakravarthy Vasudevan, Ravi Ramamurthi

**Affiliations:** Department of Neurosurgery, Voluntary Health Services, Adyar, Chennai-600 113, India

Dear Sir,

Medulloblastomas are rarely seen in the adult population, accounting to less than 1% of primary adult brain tumors.[6] It commonly arises from the cerebellar vermis.[5,6] There are only a few cases of cerebellopontine (CP) angle medulloblastomas. Most of them are intra-axial. Extra-axial site of this tumor is extremely rare.[2] Ours is the first case of metastasis from CP angle medulloblastoma.

**Figure 1:**
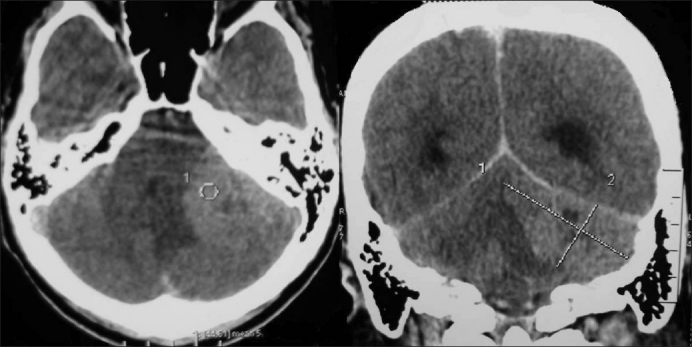
Contrast Computed tomography scan of the brain axial and coronal reconstruction showed a left CP angle mixed-density nonenhancing lesion with broad-based tentorial attachment

**Figure 2:**
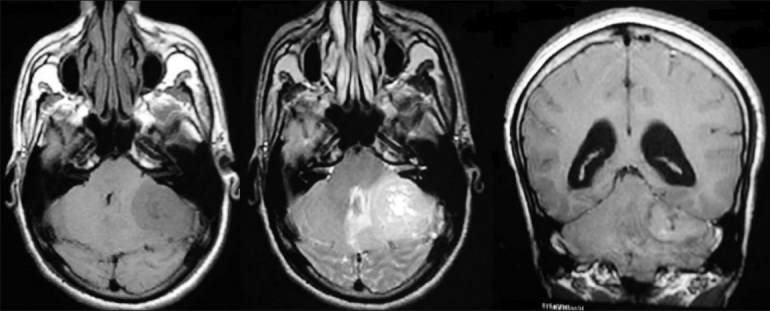
MRI of the brain axial T1, T2 and coronal sections showed a left CP angle lesion with broad-based tentorial attachment

**Figure 3:**
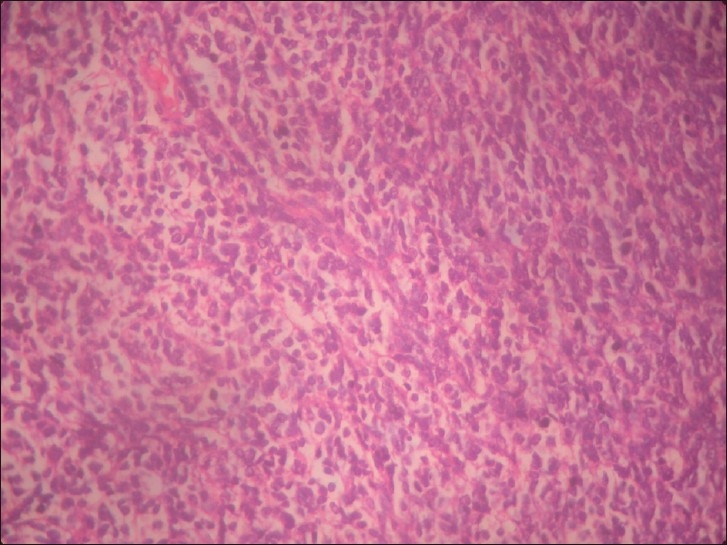
Microphotograph of the histopathology slide. (H and E, ×20). showed a highly cellular tumor composed of rosettes of small round cells, with high nucleus-cytoplasm ratio —suggestive of medulloblastoma

**Figure 4:**
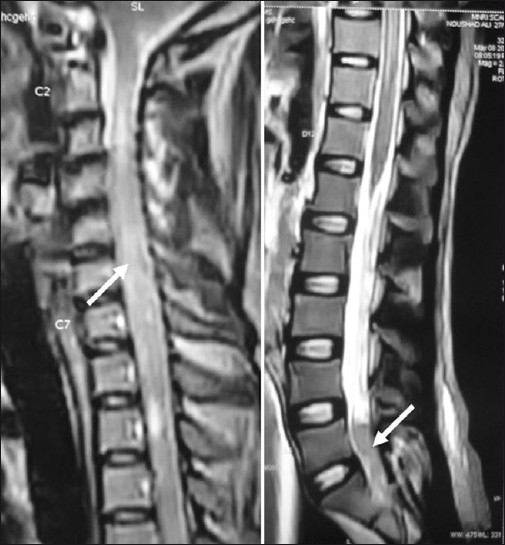
Saggital MRI of the cervical and sacral region showing drop metastasis (shown by arrows)

A 22-year-old male patient presented with headache, vomiting, ataxia, and left facial weakness of one-month duration. Vision was normal. Fundus examination showed papilloedema. He had left lower motor neuron facial paresis, left IX and X cranial nerve paresis, and left cerebellar signs. Computed tomography scan of the brain showed a left CP angle mixed-density nonenhancing lesion (5.4 × 2.8 cm) with broad-based tentorial attachment and displacement of the fourth ventricle causing obstructive hydrocephalus [Figure 1]. Findings were confirmed by magnetic resonance imaging (MRI) [Figure 2]. He had previously undergone right ventriculo peritoneal shunt for obstructive hydrocephalus elsewhere. He underwent left retromastoid craniectomy and gross total excision of the lesion. There was clear plane between the tumor and cerebellum, whereas it was adherent to tent laterally. Histopathology showed a highly cellular tumor composed of rosettes of small round cells, with high nucleus-cytoplasm ratio and increased mitotic figures—suggestive of classical medulloblastoma—WHO grade IV [Figure 3]. Postoperatively, he improved. He was advised craniospinal radiotherapy which he failed to receive and presented 15 months later with progressive quadriparesis and sensory impairment of five-month duration with bladder and bowel involvement. On examination, he had 3/5 motor power in the upper limbs and 2/5 in lower limbs and sensory impairment below D4 dermatome. All deep tendon reflexes were brisk with bilateral extensor plantar reflexes. All superficial reflexes were absent. MRI of the brain and spine showed recurrent left CP angle medulloblastoma and intramedullary lesion at cervical level and intradural extramedullary lesion at sacral level, suggestive of drop metastasis in the spine from CP angle medulloblastoma [Figure 4].

Medulloblastoma usually occur in inferior medullary velum in the midline.[6] However, rarely they may occur laterally in the cerebellar hemisphere in adults.[3] Origin of medulloblastoma may be either from germinal cells or their remnants situated at the end of posterior medullary velum or from remnants of the external granular layer.[5,7] Their development in the CPA may be from the remnants of the external granular layer in the cerebellar hemisphere, including the flocculus which faces the CP angle.[5] In CP angle medulloblastomas, though fifth, sixth, and eighth cranial nerves are frequently involved,[4] these nerves were spared in this patient. CP angle medulloblastomas are rare with nearly 35 cases published in the literature,[2,5] of which only 9 are in adults.[2,5] The lack of association with any cerebellar tissue and extra-axial location in the region of CP angle is an extremely rare phenomenon.[2] Medulloblastomas are known to metastasize through CSF to the spinal canal, leptomeninges, and supratentorial regions. Metastasis in medulloblastomas vary between 38 and 60% in various series,[1,6] with the spinal canal being the commonest site with approximately 58%.[6] Kumar *et al*. have reported a case of vermian medulloblastoma with metastasis to the CP angle.[5] To the best of authors’ knowledge, spinal metastasis from CP angle medulloblastoma has not been reported till date.

Extra-axial site of this tumor is extremely rare but must be considered in the differential diagnosis of extra-axial CP angle lesions. Any neurological deterioration seen in follow-up patient must be evaluated for metastasis.
